# Impact of Segmented Magnetization on the Flagellar Propulsion of Sperm‐Templated Microrobots

**DOI:** 10.1002/advs.202004037

**Published:** 2021-02-24

**Authors:** Veronika Magdanz, Jacopo Vivaldi, Sumit Mohanty, Anke Klingner, Marilena Vendittelli, Juliane Simmchen, Sarthak Misra, Islam S. M. Khalil

**Affiliations:** ^1^ Applied Zoology Technical University of Dresden Dresden 01069 Germany; ^2^ Smart Nano‐Bio‐Devices Group Institute for Bioengineering of Catalonia Barcelona 08028 Spain; ^3^ Department of Computer Control and Management Engineering Sapienza University of Rome Rome 00185 Italy; ^4^ Surgical Robotics Laboratory Department of Biomechanical Engineering University of Twente Enschede 7522 NB The Netherlands; ^5^ Department of Physics The German University in Cairo New Cairo 13411 Egypt; ^6^ Physical Chemistry Technical University of Dresden Dresden 01069 Germany; ^7^ Surgical Robotics Laboratory Department of Biomedical Engineering University of Groningen and University Medical Center Groningen Groningen 9713 GZ The Netherlands

**Keywords:** biohybrid microrobots, flagellar propulsion, magnetic actuation, nanoparticles, sperm cells

## Abstract

Technical design features for improving the way a passive elastic filament produces propulsive thrust can be understood by analyzing the deformation of sperm‐templated microrobots with segmented magnetization. Magnetic nanoparticles are electrostatically self‐assembled on bovine sperm cells with nonuniform surface charge, producing different categories of sperm‐templated microrobots. Depending on the amount and location of the nanoparticles on each cellular segment, magnetoelastic and viscous forces determine the wave pattern of each category during flagellar motion. Passively propagating waves are induced along the length of these microrobots using external rotating magnetic fields and the resultant wave patterns are measured. The response of the microrobots to the external field reveals distinct flow fields, propulsive thrust, and frequency responses during flagellar propulsion. This work allows predictions for optimizing the design and propulsion of flexible magnetic microrobots with segmented magnetization.

## Graphical abstract

Sperm‐templated flexible magnetic microrobots are categorized based on the location and combination of their magnetic segments. The different magnetization profiles lead to distinct wave patterns once exposed to rotating 3D magnetic fields. This results in a variety of frequency responses, flow fields surrounding the microrobots and propulsive thrust force. The article gives indications for the design of flexible magnetic microrobots.

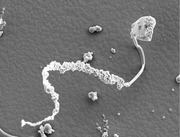

## 1 Introduction

Swimming at low Reynolds numbers is one of the main challenges in microrobotics. Nature has provided different ways to generate fluid flow and achieve drag‐based propulsion. Spermatozoa, bacteria and other motile microorganisms owe their ability to propel themselves in a viscosity‐dominated flow regime to the transverse waves generated by their self‐propelling undulatory systems.^[^ ^1^ ^]^ These natural undulatory systems are of particular interest for engineers to achieve controlled actuation when exposed to an external stimulus. In many cases the biological component serves to propel a synthetic structure by the internal active moment of the cell or serves as a structural template actuated by external stimuli.^[^ ^2^, ^3^ ^]^ These biohybrid approaches offer the opportunity not only to enhance the structural flexibility of the microrobot but also to achieve higher level of biocompatibility and degradability.

Microrobots with soft bodies provide distinct advantages as opposed to their rigid counterparts. They can adapt their shape according to the rheological properties of the fluidic environment, and have reconfigurable structures and high capability for grasping and drug delivery.^[^ ^4^, ^5^, ^6^, ^7^, ^8^ ^]^ Soft microrobots have been developed by fabricating chains of paramagnetic particles,^[^ ^9^ ^]^ magnetic rods connected with flexible hinges,^[^ ^10^, ^11^, ^12^ ^]^ hydrogels with programmable motility,^[^ ^13^, ^14^, ^15^, ^16^, ^17^, ^18^ ^]^ polymer sheets with certain magnetization profiles^[^ ^7^, ^19^ ^]^ or elastomeric jellyfish‐like robots.^[^ ^20^ ^]^ Flexible biohybrid microrobots have been proposed in the past few years using DNA strands as flagella,^[^ ^21^ ^]^ or heart cells cultured along PDMS strips.^[^ ^22^ ^]^ Another method to construct a self‐propelling undulatory system involves the integration of a motile cell or microorganism to a magnetic constituent. This biohybrid approach relies on the propulsive force of the motile microorganisms and uses the external stimuli for directional control only.^[^ ^23^, ^24^, ^25^, ^26^ ^]^ Creation of such types of biohybrid systems depends on trapping sperm cells inside magnetic microtubes or achieving bio‐adhesion between bacteria and particles.^[^ ^23^, ^27^ ^]^ It is often the case that this approach yields a limited number of functional microrobots, because the underlying process is dependent on coupling of microstructures to motile cells. Further, their operation time is fundamentally limited by the cell's viability and other environmental stimuli such as temperature, pH, and medium components.

In this work, we present several undulatory systems with segmented magnetization on the cellular segments. This functionalization led to the development of a sperm‐templated microrobot that is highly compliant and the inside of its organic body enables drug loading.^[^ ^28^ ^]^ Our undulatory system takes a similar biohybrid approach, but makes use of biological entities as templates. This method uses the cell body or biological structure to fabricate a flexible microrobot, and achieves distributed actuation and traveling wave propulsion under the influence of time‐varying external magnetic fields.^[^ ^29^, ^30^ ^]^ One desirable property of this type of undulatory system is their naturally optimized design and elasticity as opposed to their artificial counterparts. Moreover, their response is dependent on the robustness of the magnetic torque that is exerted on their magnetic dipole, and their operation time is not limited by physiological environments or the lifetime of the cell. Recently, spermatozoa were self‐assembled with magnetic nanoparticles and the cells were loaded with anticancer drugs. This functionalization enables magnetic actuation and produces detectable ultrasound waves necessary to localize swarms of sperm cells.^[^ ^28^ ^]^

**Figure 1:**
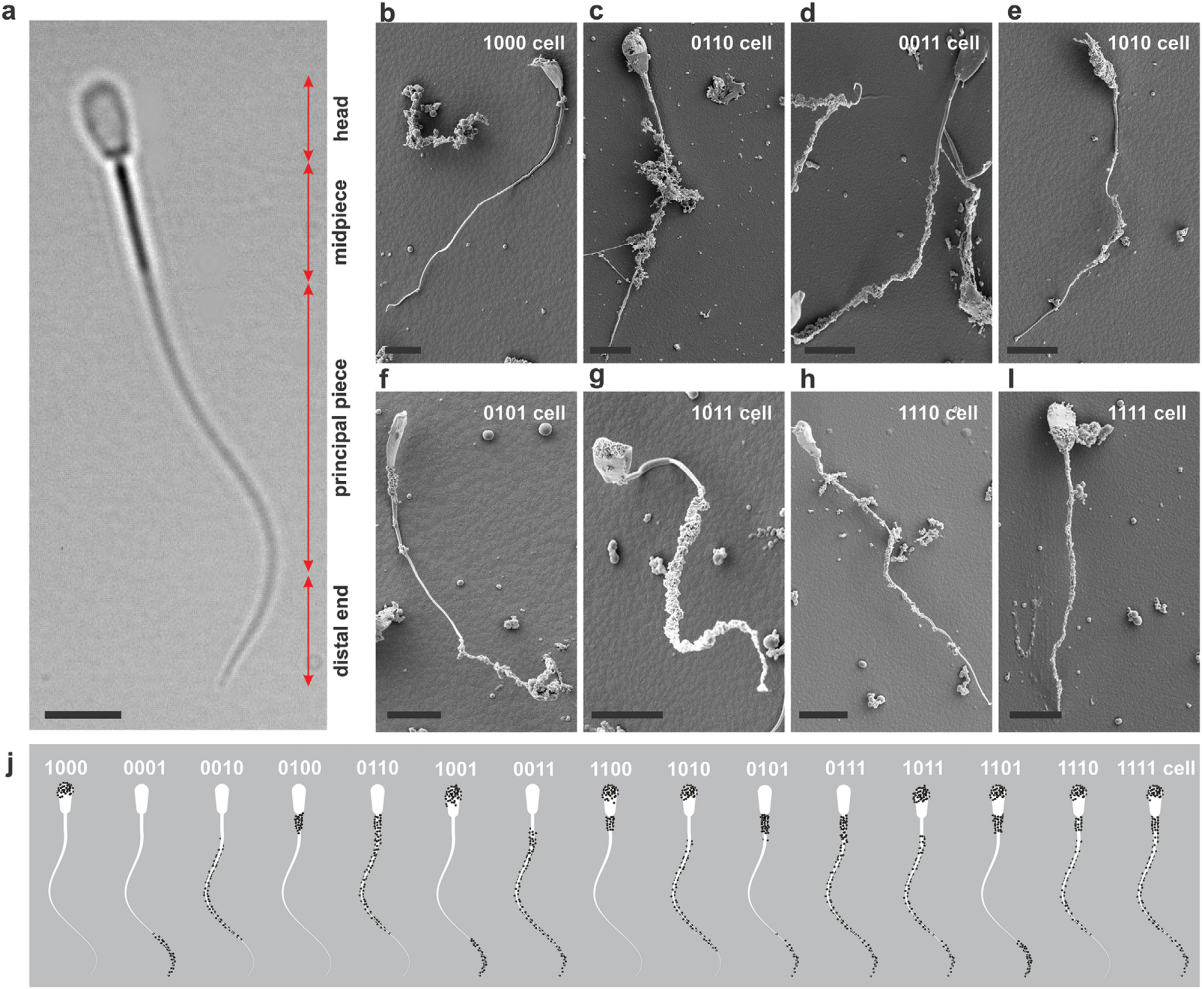
Sperm‐templated microrobots are sorted into categories based on the magnetized cellular segment. a) Bovine sperm cell consists of a 10‐µm‐long, 5‐µm‐wide, and 1‐µm‐thick flat head, 13‐µm‐long midpiece, 40‐µm‐long principal piece and 7‐µm‐long distal end. b–i) Scanning electron microscopic images of eight representative different categories of microrobots. Scale bar 10 µm. Maghemite nanoparticles adhere to different segments along the sperm cell resulting in different magnetization profiles along the sperm cell. b) 1000 cells. c) 0110 cells. d) 0011 cells. e) 1010 cells. f) 0101 cells. g) 1011 cells. h) 1110 cells. i) 1111 cells. Scale bars 10 µm. j) Overview of all possible configurations of sperm‐templated microrobots depending on attachment of magnetic nanoparticles to the segments of a bovine sperm cell.

We investigate the design features of sperm‐templated microrobots with segmented magnetization by analyzing the distributed actuation of all possible configurations that are obtained by self‐assembly of positively charged nanoparticles and negatively charged bovine sperm cells. These biohybrid microrobots are classified into distinct categories based on the magnetized cellular segments (head, midpiece, principal piece, and distal end), as shown in **Figure** **1**a, and investigated to realize the crucial properties for propulsion.

## 2 Categorization of Sperm‐Templated Microrobots by Segmented Magnetic Regions

A desirable approach for magnetizing the passive flagellum is to cover its surface with nanoparticles, resulting in an organic body with agglomerated magnetite particles. Even with this surface coating, loading the inside of the cell with nanoparticles through fluid phase endocytosis is possible,^[^ ^31^ ^]^ but it is not likely to improve the magnetization of the segment owing to the low amount of internalized nanoparticles compared to coated nanoparticles.

The surface charge of the cell dictates the location of the agglomerates and varies across cells, leading to distinct magnetization profiles along the flagellum (Figure 1). The net surface charge between the cell and the nanoparticles and their spatial arrangement are important factors in the determination of the size and location of the agglomerates along the cell. The net negative surface charge is indicative of the total amount of positively charged nanoparticles attached to the cell, whereas the spatial arrangement of the negative cell charges influences the nanoparticle location. Therefore, the amount of the positively charged particles on each cellular segment is dependent on their net negative charge. Consider, for example, a cell surrounded by a fluid with homogenous distribution of nanoparticles. The long‐range Coulomb forces will attract the nearest particle to the cell and uniform coating is likely to occur. In this case, the cell would have a uniform coating, and it is referred to as 1111 cells. An alternative scenario, which is realistic when the nanoparticles agglomerate and form clusters, would result in nonuniform distribution of nanoparticles along the cell and distinct agglomerated magnetization profile.

Possibilities range from relatively large agglomerates along the length to several small clusters distributed over several locations. The nonuniformity of the surface charge and the distribution of the nanoparticle cluster vary during the electrostatic‐based self‐assembly,^[^ ^28^ ^]^ allowing us to obtain agglomerates at one segment for example, as shown in Figure 1b (1000 cell). The four representative samples in Figure 1c–f contain two particles agglomerates along two cellular segments, and they are referred to as 0110, 0011, 1010, and 0101 cells. Similarly, the two samples in Figure 1g,h, for example, exhibit particles all over the cell with the exception of the midpiece and the distal end, and designated as 1011 and 1110 cells, respectively. Finally, Figure 1i presents a sample with particles agglomerates at all cellular segments, which we refer to as 1111 cell. Clearly, magnetic nanoparticles are electrostatically self‐assembled around the cell to achieve distinct magnetization profiles, and understanding the cell biology is only one of the many ways to explain why particles adhere to various cellular segments.

The evolution of spermatozoa has led to the male gametes being the most diverse cell type in terms of size, morphology, and function.^[^ ^32^, ^33^ ^]^ This diversity has evolved as a consequence of the highly variable conditions under which spermatozoa compete to fertilize, leading to sexual selection processes that are governed by the conditions in the male and female reproductive organs. The sperm diversity is large within an ejaculate, within a species and across species; this is the case because, due to the selective pressure in reproduction and because sperm cells have adapted to their specific fertilization environment. The underlying selection mechanisms for sperm diversification are addressed by a number of evolutionary biology studies.^[^ ^34^ ^]^ The natural diversity of sperm cells also displays multimodal propulsion (e.g., the ability to move backward or forward or to change direction in response to various external stimuli). This diversity has inspired researchers to design and fabricate different types of biohybrid microrobots.

As previously mentioned, the surface charge of spermatozoa varies throughout the cell and also during the differentiation and maturation of sperm cell.^[^ ^35^, ^36^ ^]^ The surface charge of the cell is an important factor in the determination of the magnetization profile of the sperm‐templated microrobots. Magdanz et al. have demonstrated that bovine sperm cells show nonuniform charge distribution and negative overall net charge.^[^ ^37^ ^]^ Thus, coating the negatively charged areas along the cell with positively charged particles allows various cellular segments to be selectively magnetized. This self‐assembly process between the negatively charged bovine spermatozoa and positively charged iron oxide particles results in various configurations (see Figure 1), that can be sorted into different categories based on the location of the magnetized segment along the following four segments: the flat 10‐µm‐long, 5‐µm‐wide, and 1‐µm‐thick head; the mitochondrial sheath surrounded midpiece located adjacent to the head with relatively high stiffness; the principal and most flexible piece of the tail; and the distal end (see Figure 1a). If we consider these four distinct segments of the cell body, and that each segment can either contain magnetic particles (**m**_*i*_ ≠ 0) or not ( **m**_*i*_ =  0), we obtain fifteen categories, where **m**_*i*_ is the magnetization of the *i*th segment along the cell. The eight samples in see Figure 1b–I, for example, contain particles on different cellular segments. All samples were fabricated by means of electrostatic self‐assembly using the same amount of concentrated nanoparticle suspension (50 µL) and a vial of sperm cell suspension (1 mL). This mixture generated fifteen categories of sperm‐templated microrobots within one sample (see the Experimental Section).

The likelihood of having any of these fifteen categories depends on many factors. The first is the nonuniform charge distribution along the surface of the sperm cells. The sperm head contains the acrosomal area which is important for oocyte fusion, while the midpiece carries many mitochondria. The second is the different types of proteins, glycoproteins, and lipids within the membrane of the cells. Both factors affect the number of each category per sample. However, the surface charge also changes during the lifetime of a cell, and is dependent on its developmental state and quality.^[^ ^37^ ^]^ Therefore, separation of sperm by their net surface charge has been proposed as a method for in vitro sperm selection.^[^ ^38^, ^39^ ^]^ Typically, a sample contains sperm cells with various surface properties. Figure 1b–i display scanning electron micrographs of some categories. All configurations displayed in Figure 1b–i are frequently encountered with the exception of cells with uncoated heads. The surface area of the head is locally greater than that of any other part of the flagellum, making the possibility of samples with uncoated heads relatively low with a likelihood of 7% for 0001, 0010, 0011, 0101, 0110, or 0111 cells (see the Experimental Section). In contrast, cells with coated heads are likely to form and our characterization measurements show that the likelihood of forming 1111, 1011, and 1110 cells by electrostatic self‐assembly are 37.7%, 22%, and 11%, respectively. Finally, 1010 and 1000 cells have a likelihood of 6% and 12%, respectively. Therefore, associated with the decrease in the surface area of segments along the cell is a decrease in the likelihood of having cells with uncoated heads. At the same time, the total amount of magnetic particles per sperm varies between the categories. The highest amount is found on the 1111 cells with 9  × 10^−10^ g per cell, followed by the microrobots with three magnetic segments containing an average of 8.8  × 10^−10^ g per cell, the category with two magnetic segments with 5  × 10^−10^ and 3.1  × 10^−10^ g per cell for microrobots with one magnetic segment.

## 3 Passively Propagated Wave Along Flagellum with Multiple Particle Agglomerates

**Figure 2:**
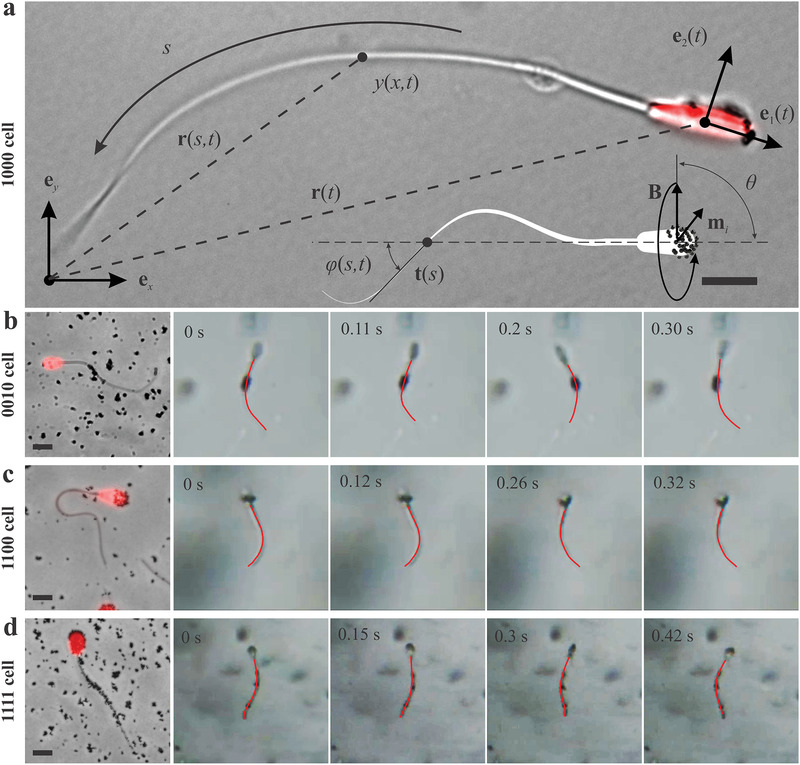
Sperm‐templated microrobot consisting of an organic body and magnetizable nanoparticles. Magnetic torque initiates transverse bending waves along the passive flagellum. a) The dead sperm head is fluorescently labeled with propidium iodide. The flagellum is characterized by the tangent angle (*φ*) that is enclosed between the local tangent (**t**) along the arc length *s* and axis **e**_1_ at position **r**(*s*, *t*). The deformation along the flagellum is described with respect to the material frame of reference (**e**_1_,**e**_2_) of the head, and **r**(*t*) is the position vector of the center of the head. The induced magnetization **m** *_i_* of the *i*th magnetized segment along the flagellum trails behind the actuating magnetic field **B**, at angular velocity ω**e** *_x_*. Scale bar 5 µm. b–d) Three representative sperm‐templated microrobots and the respective image sequences of their motion. Scale bars 10 µm. b) 0010 cells swim at 6.5 ± 4.6 µm s^−1^ (*f*  =  5 Hz). d) 1100 cells swim at 4.6 ± 4.7 µm s^−1^ (*f*  =  4 Hz). d) 1111 cells swim at 3.5 ± 0.3 µm s^−1^ (*f*  =  3 Hz).

We consider a sperm cell with nanoparticle clusters attached along its length (see **Figure** **2**) and predict the fluid response during its flagellar propulsion.

### 3.1 Theoretical Model

To produce flagellar propulsion, we must generate a magnetization which is dependent on the applied magnetic field, **B**. This magnetization initiates magnetic torque and transverse bending waves along the flagellum, which is characterized by the tangent angle *ϕ*(*s*, *t*) enclosed between the local tangent (***t***) and the head axis **e**_1_ along the arc length *s*, and the amplitude *y*(*x*, *t*) that is measured in the frame of reference (**e** *_x_*, **e** *_y_*), as shown in Figure 2a. The field magnetizes the clusters to a magnetization **m** *_i_*. The distance between these segments is small compared to any change in the actuating magnetic field. Therefore, the magnetization of each segment is dependent on the same field, **B**, and trails behind when it periodically oscillates under the action of the following magnetic torque^[^ ^40^ ^]^

(1) $$\left|T_{i}\right|=\frac{v_{i}\left|n_{r}-n_{a}\right|}{2n_{a}n_{r}\mu_{0}}\;{\left|B\right|}^{2}\sin\left(2\theta \right)$$

where **T** *_i_* is the magnetic torque exerted on the *i*th segment along the cell, and *v_i_* is the volume of its magnetic material. Further, *n*_a_ and *n*_r_ are demagnetization factors along the axis of symmetry and radial direction of the nanoparticle cluster, respectively, which characterize the magnetic susceptibility and the relative permeability. *μ*_0_ is the permeability of free space and *θ* is the angle between the local tangent along *s* (at the position of the cluster) and external magnetic field (Figure 2a). Figure 1 shows that each segment along the length can include a cluster of nanoparticles. Therefore, the magnetic field can exert multiple magnetic torques simultaneously to achieve transverse bending at different locations along the length. Oscillation of the magnetic field also leads to planar travelling waves along the flagellum, allowing the microrobot to swim using planar flagellar propulsion. Regardless of the pattern of the field (planar oscillating or rotating field), the resulting transverse deformation of the flagellum will propel the cell in low Reynolds number (*Re*), as shown in Figure 2b–d for three configurations of sperm‐templated microrobots (0010, 1100, and 1111 cells).

When the magnetic field is applied, the 0010 cell is magnetized at its principal piece, leading to a bent region along the passive flagellum where particles adhere to the cell. Figure 2b demonstrates how a rotating magnetic field about the propulsion axis **e** *_x_* can be used to propel the cell in low‐*Re*. The bent region trails behind the rotating field and initiates transverse bending waves near the middle of the cell. Because the proximal and distal ends of the cells are free, the amplitude near the free ends becomes progressively smaller. Other types of microrobots use the same rotating field to propel in low‐*Re*. Figure 2c shows the flagellar propulsion of 1100 cell. In this case, the bent region at the head and midpiece initiates bending waves toward the distal end, and consequently, the cell moves in the direction opposite to the traveling wave. Therefore, each type produces a particular bending wave pattern, based on the location of the magnetized cellular segment along the length of the flagellum.

The compliant flagellum can exhibit planar or helical travelling waves when the field oscillates in‐plane or rotates about the axis of symmetry of the cell (**e**_1_). These fields enable the microrobot to have comparable flagellar propulsion with that of live sperm cells through a propagation of waves that is governed by two sets of forces.^[^ ^41^ ^]^ The first is the elastic forces (*F*_el_) which tend to restore the natural shape of the flagellum and is given by^[^ ^42^ ^]^

(2) $$dF_{\mathrm{el}}=\;\kappa \frac{\partial^{4}y\left(x,t\right)}{\partial x^{4}}dl+\frac{\partial^{2}M\left(x,t\right)}{\partial x^{2}}dl$$

where d*F*_el_ is the elastic force on an element dl and *κ* is the bending stiffness of the flagellum. The distribution of bending moment *M*(*x*, *t*) along the flagellum describes the active bending generated by the magnetized segment when the external magnetic field is applied and periodically varied. The second is that of the viscous forces (*F*_visc_) which opposes the motion and is calculated as follows^[^ ^43^ ^]^

(3) $$dF_{\mathrm{visc}}=\;\xi_{⊥}\;\frac{\partial y\left(x,t\right)}{\partial t}dl$$

where *ξ*_⊥_ is the normal drag coefficient, and d*F*_visc_ is linearly proportional to the velocity of the segment dl due to the low‐*Re* characteristic of the flagellar propulsion in a fluid with viscosity *η*. The elastic (2) and viscous (3) forces can only influence a segment along the flagellum owing to the low‐*Re* characteristics of the medium and absence of other external forces such as magnetic forces (uniform field is used for actuation) and interactions. Therefore, the elastic force on a segment dl must equal the viscous force, and by combining Equations (2) and (3), we have

(4) $$\kappa \frac{\partial^{4}y\left(x,t\right)}{\partial x^{4}}+\;\frac{\partial^{2}M\left(x,t\right)}{\partial x^{2}}=\;\xi_{⊥}\;\frac{\partial y\left(x,t\right)}{\partial t}$$

The steady‐state solution of Equation (4) characterizes the flagellar deformation. The flagellar beat pattern of the microrobot can also be reconstructed as^[^ ^43^, ^44^ ^]^

(5) $$r\left(s,t\right)=\;r\left(t\right)-ae_{1}\left(t\right)-\int_{0}^{s}\left(\cos\varphi \left(\nu ,t\right)e_{1}+\sin\varphi \left(\nu ,t\right)e_{2}\right)d\nu$$

where **r**(*s*, *t*) is the position vector of flagellum centerline with respect to the material frame of reference (**e**_1_(*t*),**e**_2_(*t*)). Further, **r**(*t*) is the position of the center of the sperm head and 2*a* is its major diameter (see Figure 2a). As the magnetic field rotates about **e** *_x_*, the clusters are instantaneously magnetized and trails behind the field, causing the flagellum to deform. This is the method used to propel the cell forward by pushing the fluid backward through wireless magnetic actuation and the following average thrust force

(6) $$F\;=\langle \int_{0}^{L}\left[\xi_{⊥}U_{⊥}\left(s,t\right)+\xi_{∥}U_{∥}\left(s,t\right)\right]dl\rangle$$

where **U**_⊥_(*s*,*t*) and **U**_∥_(*s*,*t*) are the normal and tangential velocity components on the segment dl, respectively (see the Experimental Section) and *ξ*_∥_ is the tangential drag coefficient. Each segment dl along the length (*L*) of the flagellum pushes laterally against its surrounding by drag force *ξ*_⊥_ **U**_⊥_(*s*,*t*) and parallel to its length with drag force *ξ*_∥_ **U**_∥_(*s*,*t*). Therefore, for a given propagating wave along the flagellum, the average thrust force is determined over the length and one period of oscillation, where 〈 · 〉 denotes averaging over one period of oscillation. This force completes the relation between the magnetic actuation of a particular magnetic segment along the flagellum and the generated propulsive thrust.

### 3.2 Fluid Response for Various Segmented Magnetization

The fluid response is dependent on the generated traction forces along the flagellum and the swimming speed under the influence of the rotating magnetic field. The regularized Green's function for Stokes flow provides the fluid response as the superposition of *N* responses due to *N* spread‐out point forces^[^ ^45^ ^]^

(7) $$\left[\begin{array}{c} U_{∥}\left(r_{0},t\right)\\ U_{⊥}\left(r_{0},t\right) \end{array}\right]=\frac{1}{8\pi \eta }\;\sum_{n\;=\;1}^{N}\sum_{k\;=\;1}^{3}S_{k}^{\varepsilon }\left(r_{n},r_{0}\right)\left[\begin{array}{c} f_{n,k}^{∥}\\ f_{n,k}^{⊥} \end{array}\right]A_{n},$$

where **U**_∥_(**r**_0_,*t*) and **U**_⊥_(**r**_0_,*t*) are tangential and normal velocities of the segments at point **r**_0_ along the flagellum or any point in fluid, resulting from the superposition of *N* tangential and normal forces, $f_{n,k}^{∥}$ and $f_{n,k}^{⊥}$. The regularized Green's function $S_{kn}^{\varepsilon }$ depends only on the relative distance between the observation point of the velocity and application point of the force, *ε* determines the amount of spread‐out for the superposition, and *A_n_* is the quardature weight of the *n*th application point. In this regularized Stokeslets theory, the tangential and normal velocities are known based on the kinematics of the flagellum at any point along the arc length *s*. The tangential, $U_{∥}\;\left(s,t\right)=\left(\dot{r}\left(s,t\right)\cdot t\left(s,t\right)\right)\;t\left(s,t\right)$, and normal, $U_{⊥}\;\left(s,t\right)=\dot{r}\;\left(s,t\right)-U_{∥}\left(s,t\right)$, components are determined directly from the balance between the elastic and drag forces given by Equations (1) and (2). Under magnetic actuation, each segment along the flagellum is moving transversely across the long axis of the head **e**_1_, leading to flagellar swim.

**Figure 3:**
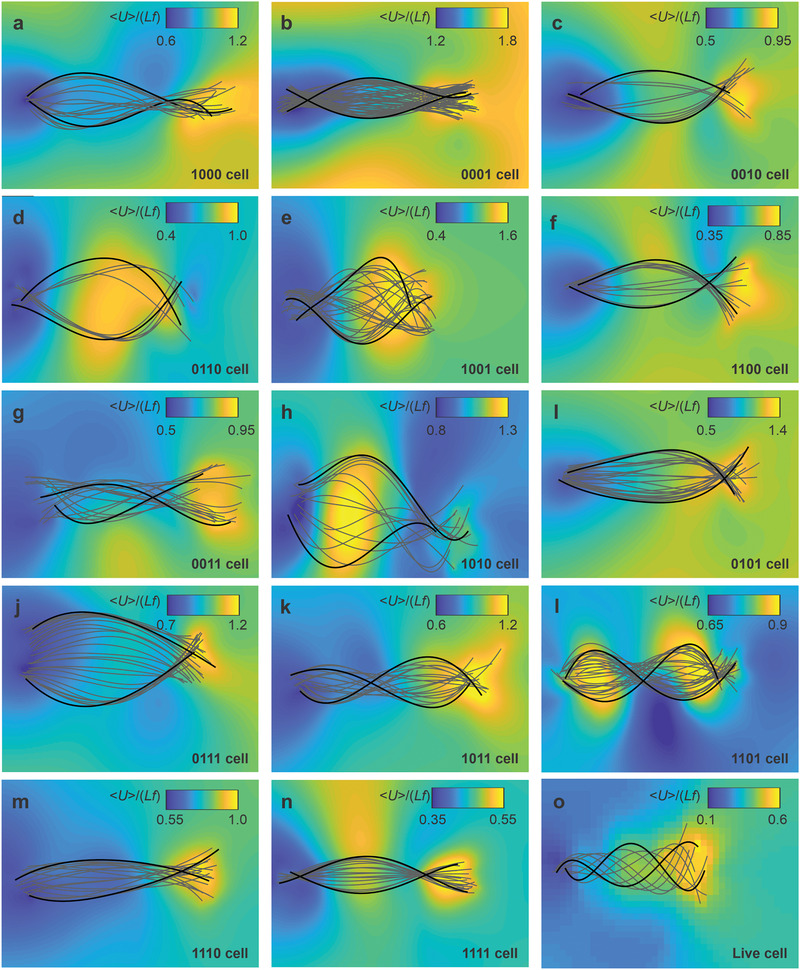
Average flow field is calculated using the measured wave pattern of the sperm‐templated microrobots over one complete beat cycle. The dimensionless 〈*U*〉/*Lf* is calculated using Equation (7) based on the measured flagellar beat of each microrobot. Envelopes of the wave patterns are indicated by the black lines. a–c) The microrobots are actuated using a single magnetic segment along the length. d–i) Two magnetic segments along the length. j–m) Three magnetic segments along the length. n) Four magnetic segments along the length. o) Flow field around a live sperm cell without magnetic particles. See Videos S1–S4 in the Supporting Information for illustration of the flow fields.

**Figure** **3** demonstrates the average flow fields of the surrounding fluid during a complete beat cycle (see the Experimental Section and Figure S1 (Supporting Information)). Each configuration is actuated, and the flagellar bending waves are measured and characterized using Equation (5) based on the tangent angle along the arc length. The velocity of each flow field is normalized by the length and frequency (*Lf*). The flow field increases toward the distal end of the cell when the bending wave is initiated at the head (1000 cell), as shown in Figure 3a. The actuation of the head in this configuration leads to an exponential decay of the bending amplitude along the flagellum. Therefore, we attribute the increased flow field to the rate of wave propagation which increases as the flagellum taper over the length. Type 0001 cell is also actuated at one of its free ends (distal end), as shown in Figure 3b. However, actuation of the distal end results in higher velocities of the flow fields, while the difference between the velocities at the free ends has the same magnitude.

Like 1000 and 0001 cells, actuation of the principal piece results in relatively higher flow fields at the distal end (Figure 3c). Therefore, a desirable approach for actuating a passive filament would be to magnetize one segment along the filament and initiate the transverse bending waves to obtain increasing flow field in the direction of distal end. The flow fields of type 0010 cell are symmetric with respect to the propulsion axis (**e** *_x_*), as shown in Figure 3c. Therefore, microrobots of this type are likely to achieve flagellar propulsion with relatively high linearity of the taken pathway. The flow field of the 1000 cell shows notable asymmetry with respect to **e** *_x_* and **e** *_y_* and will likely exhibit low linearity, leading to reduced net propulsion.

Figure 3d–i show the fluid response for microrobots with two magnetic segments. The presence of particles agglomerates near the middle (0110 cells) of the flagellum produces low flow fields at the free ends (see Figure 3d). Unlike 0110 cells, the behavior of 1001 cells indicates that the flow field is low near the head in the region of maximum stiffness. The flow field is highly symmetric with respect to the propulsion axis ***e**_x_* compared to 0110 cells, as shown in Figure 3e. When particle agglomerates are located at the head and midpiece (see Figure 3f), we observe a similar behavior to that of type 1000 cells with more symmetric average flow field about the axis **e** *_x_*. Again, like the case of 1000 and 0010 cells, the flagellar beat patterns of 1100 and 0011 cells are quite different, leading to distinct flow fields. Figure 3g shows high velocity near the distal end and symmetric flow field about **e** *_x_*. Figure 3h,i demonstrates the beat patterns and flow fields when the two magnetic segments are separated by free regions. The average velocity is notably high for type 1010 (see Figure 3h) cells compared to 0101 cells (see Figure 3i) only near the middle of the flagellum. Type 1010 cells are very similar to 0010 and 0011 cells in that both have high flow field near the distal end and near the middle of the principal piece. These types generate flow fields with two local maxima at locations where the amplitude is greater than that of any other part along the length of the flagellum, as shown in Figure 3c,g,h. However, the amplitude of type 1010 cells has its maximum value not near the free distal end but near the midpiece. Therefore, the location of the maximum flow field is opposite to that of types 0010 and 0011 cells.

When the number of particle agglomerates is increased to three segments, the behavior of the microrobot becomes increasingly dependent on the location of the nonmagnetic region. Figure 3j–m demonstrate a flagellar beat with constant curvature for types 0111 and 1110 cells. In these cases, the magnetic segments are not separated by nonmagnetic regions and the mean flagellar curvature is constant. Both type 1011 and 1101 cells exhibit curvatures with opposite signs, as shown in Figure 3k,l, respectively, leading to two locations where the amplitude is greater than that of any other part of the cell. These two types also produce flow fields with two local maxima at the locations of the maximum amplitude. Finally, the behavior of the microrobot approaches continuous actuation when particle agglomerates adhere to all segments. In this case, the flagellar beat pattern produces uniform flow field with a velocity slightly higher near the middle and the free distal end of the flagellum, as shown in Figure 3n. However, the amplitude of the bending waves is reduced, resulting in lower values of the average flow fields compared to all other microrobots.

In contrast to magnetically actuated microrobots, the adenosine triphosphate (ATP) driven live sperm cells exhibit flagellar propulsion and produce flow fields with gradually increasing velocity toward the free distal end of the flagellum (see Figure 3o). This pattern can be qualitatively resembled by 1000 and 1110 cells. We can also gain additional insights into the difference between ATP‐ and magnetically‐driven microrobots using wave pattern analysis.

## 4 Wave Propagation and Propulsive Thrust of Various Segmented Magnetization

When the passive flagellum is actuated by a periodic magnetic torque the distribution, *M*(*x*), of bending moment affects the resulting wave pattern, as shown in Figure 3. Except for a few microrobots, the wave patterns have very different envelopes of motion indicated by the black curves. Each pattern is characterized by curvature/amplitude/wavelength combination to provide additional insights into the impacts of the magnetic distribution along the flagellum. To determine this combination, we use the Fourier analysis of the wave pattern (see the Experimental Section and Figure S2a (Supporting Information)) and extract the mean flagellar curvature (*K*_0_), bending amplitude (*A*_0_), and wavelength (*λ*). These wave parameters are calculated by the Fourier decomposition of the calculated tangent angle, *φ*(*s*, *t*). Since the wave variables kinematically prescribe the deformation of the passive flagellum, we also calculate the propulsive thrust for each case using Equation (6).

### 4.1 Sperm‐Templated Microrobots with a Single Magnetic Cellular Segment

In the case of a single nanoparticle cluster along the cell, the microrobots are classified into four distinct groups. All have a single magnetic segment. Therefore, transverse bending waves originate from one point of actuation. Let us begin by considering a sperm cell covered at the head only (1000 cells). In this case, the induced magnetic moment at the head trails behind the rotating magnetic field, leading to rotation about **e** *_x_*. The distant parts from the head are not influenced by magnetic torque and lag the motion of the head based on the elastic properties of the flagellum and the viscous forces of the fluid. Similar to 1000 cells, the magnetic actuation of cell covered at the distal end 0001 enables it to rotate in sync with the applied field. The distant cellular parts are not magnetized and lag the distal end. The qualitative behavior of these two microrobots is similar as the transverse bending waves initiate and propagate either from the proximal to the distal end or from the distal to the proximal end of the flagellum.

**Figure 4:**
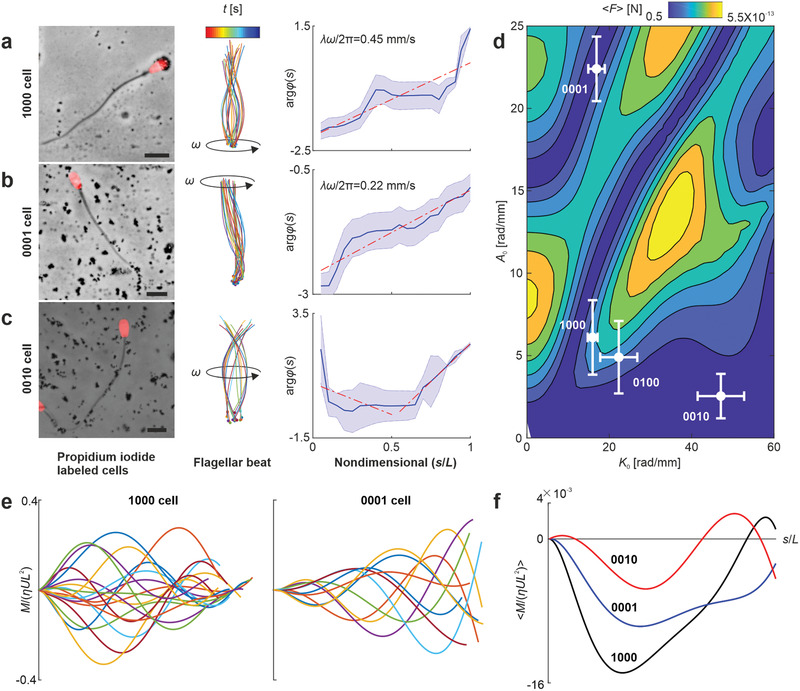
Sperm‐templated microrobots with a single magnetic segment. a–c) overlayed brightfield and fluorescent images of the microrobots with red fluorescent sperm heads (left column), wave pattern over one beat cycle (middle panel) and rate of wave propagation (arg*φ*(*s*)) over tail length (right panel). a) For 1000 cells, wave propagates from the head to the distal end with wavelength of 150 µm. b) For 0001 cells, wave propagates from the distal end to the head with wavelength of 221 µm. c) For 0010 cells, waves propagate along opposite directions toward the head and the distal end. Scale bar 10 µm. d) Time‐averaged propulsive thrust *<F>* of the flagellum is calculated using RFT (see the Experimental Section) over mean flagellar curvature, *K*_0_, range of 0–60 rad mm^−1^, amplitude rise, *A*_0_, of 0–25 rad mm^−1^, and average wavelength of 305 ± 224 µm. e) Nondimensional bending moment *M*/(*ηUL* ^2^) is calculated using the force balance between (2) and (3) based on the measured wave pattern for two representative cases of 1000 and 0001 cells. f) Nondimensional time‐averaged bending moment 〈*M*/(*ηUL* ^2^) 〉 is calculated over one beat cycle, where *η*, *U*, and *L* are the viscosity of the medium, velocity and length of the flagellum. See also Video S5 (Supporting Information) of the sperm‐templated microrobots with one magnetic segment.

**Figure** **4**a,b shows the rate of wave propagation along the length in the case of boundary actuation of 1000 cells and 0001 cells, respectively. The transverse waves propagate at speed of *λω*/(2*π*) of 0.45 and 0.22 mm s^−1^ for 1000 and 0001 cells, respectively. The difference in wave propagation rate is attributed to the elastic properties of the distant part from the actuated boundary. Actuation of the head enables the wave to propagate along the principal piece and the distal end, while rotation of the distal end enables the waves to propagate along the principal piece, midpiece and the head. The elastic properties of these parts of the cells are different as the bending stiffness decreases with the length. The flagellum tapers over the length and the stiffness decreases toward the distal end. Therefore, in the case of 1000 cells, the wave propagates along the direction of decreasing stiffness, while for 0001 cells the propagation is along the direction of increasing stiffness.

Now consider the case where the cell is covered with particles at the principal piece (0010 cells) and the proximal and distal ends are free from external force and torque. In this case, waves initiate near the middle of the cell and propagates along opposite directions toward the head and the distal end. Figure 4c shows the rate of wave propagation along the flagellum for 0010 cell. A change in the sign of the rate of wave propagation is exhibited along the flagellum and indicates the direction of the transverse bending waves. The rate of wave propagation is indicative of the direction of propulsion which occurs in the direction opposite to that of the propagating wave. Therefore, 0010 cells generate propulsive forces along opposite directions making the surface‐coating of only the principal piece undesirable in sperm‐templated microrobot design (see Video S5 (Supporting Information)).

Figure 4d shows the calculated propulsive thrust using Equation (6) based on the measured *K*_0_, *A*_0_, and *λ* for the four microrobots with single magnetic cellular segment. The flagellar pattern of the microrobots can be greatly influenced by the location of the magnetized segment along the flagellum, leading to relatively higher average thrust force for 1000 cells compared to 0010 cells. Similar to the fluid response, we use the measured wave pattern to determine the time‐averaged bending moment along the passive flagellum. Figure 4e shows the normalized bending moment, *M*/(*ηUL* ^2^), of type 1000 and 0001 cells for each measured time‐dependent deformation. Figure 4f demonstrates the time‐averaged bending moment over a complete beat cycle for the microrobots with single magnetic segment. In all cases, the maximum bending moment occurs near the middle of the flagellum, and consequently maximum transverse displacement at the same point.

### 4.2 Sperm‐Templated Microrobots with Two Magnetic Cellular Segments

**Figure 5:**
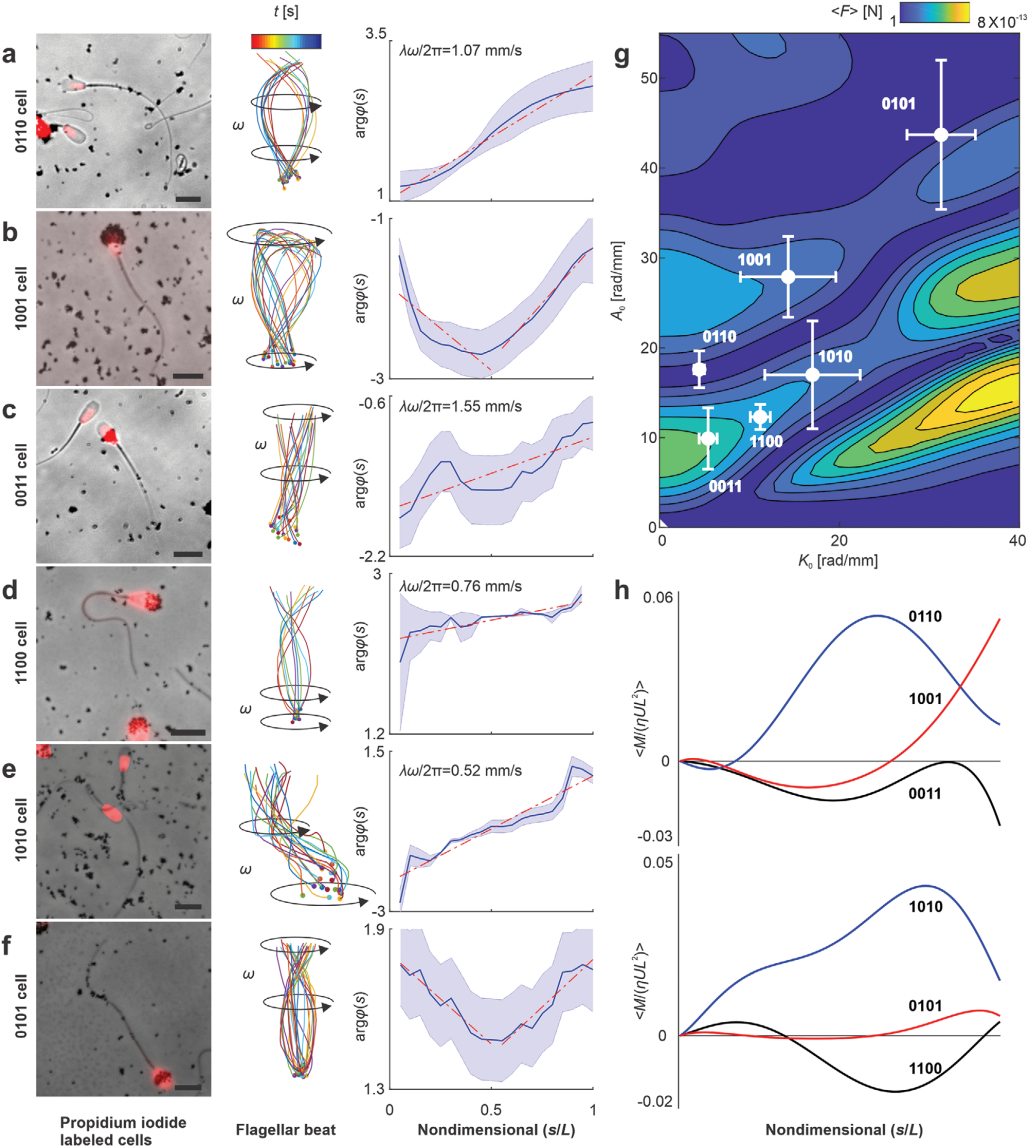
Sperm‐templated microrobots with two magnetic segments. a) 0110 cells. b) 1001 cells. c) 0011 cells. d) 1100 cells. e) 1010 cells. f) 0101 cells. Scale bar 10 µm. g) Time‐averaged propulsive thrust of the flagellum is calculated using RFT (see the Experimental Section) over mean flagellar curvature range of 0–40 rad mm^−1^, amplitude rise of 0–55 rad mm^−1^, and average wavelength of 284 ± 189 µm. h) The dimensionless time‐averaged bending moment 〈*M*/(*ηUL* ^2^) 〉 indicates location along the length of the passive flagellum where the maximum amplitude occurs due to maximum bending moment. See also Video S6 in the Supporting Information.

The heterogeneity of the surface charge along the cell enables surface‐coating along several segments. Therefore, there is another set of possible microrobot designs with two magnetic segments (see Video S6 in the Supporting Information). 0110 cells consist of magnetic elements along the midpiece and the principal piece. Even without a magnetic element on the head, the rigid connection between the midpiece and the head enables the proximal end of the cell to rotate in sync with the actuating field. **Figure** **5**a shows the rate of wave propagation along the flagellum for 0110 cell with a wavelength of 179 µm. The magnetic elements of the adjacent segments of the cell enable the transverse bending waves to propagate along one direction, as shown in Figure 5a, making them desirable in design as the propulsion occurs in the direction opposite to the wave.

Now consider the opposite case where the cell is attached to particles at the head and the distal end only. The proximal and distal ends of these 1001 cells are magnetically actuated and the transverse bending waves are initiated on the left and right sides of the cell. Figure 5b shows positive and negative rates of propagation along the filament, and thereby producing thrust forces along opposite directions. With the exception of the direction of wave propagation, 1001 cells are similar to 0010 cells as the two sets of thrust forces act in opposite directions. Therefore, it is unlikely that 1001 and 0010 cells can be effectively actuated to swim in low‐*Re*. Additional two possible categories of microrobots with two magnetic segments include 0011 and 1100 cells (Figure 5c,d). Once actuated, the distal end of the 0011 cells rotates in sync with the magnetic field while the proximal end lags the motion of the magnetized cellular segment. 1100 cells exhibit a similar behavior to 0011 cells, and once actuated the distal end lags the motion of the actuated proximal end.

The last two possible microrobots with two magnetic elements are the 1010 (see Figure 5e) and 0101 cells (see Figure 5f). There is no notable difference between the actuation of the proximal end through the head or the midpiece due to the relatively high stiffness of this part of the cell. However, the position of the second magnetized segment has a profound influence on the wave propagation, as shown in Figure 5e,f. As a result of the presence of magnetized segment on the distal end for 0101 cells, the bending waves exhibit positive and negative rates of propagation along the flagellum. The magnetized segment of the principal piece for 1010 cells enables the propagating waves to be in phase.

Figure 5g shows the net propulsive thrust of each type and indicates that 0011 cells produce maximum thrust and expected to swim at maximum swimming speed compared with other types. The bending moment along the passive flagellum of these microrobots reveals insights into the relation between stiffness and the magnetic segment. 0110 cells are actuated near the middle of the flagellum and the maximum displacement occurs near the distal end which is more flexible than any other part of the flagellum (see Figure 5h), and similarity the maximum amplitude occurs at the distal end for 1001 cells.

### 4.3 Sperm‐Templated Microrobots with Three Magnetic Cellular Segments

**Figure 6:**
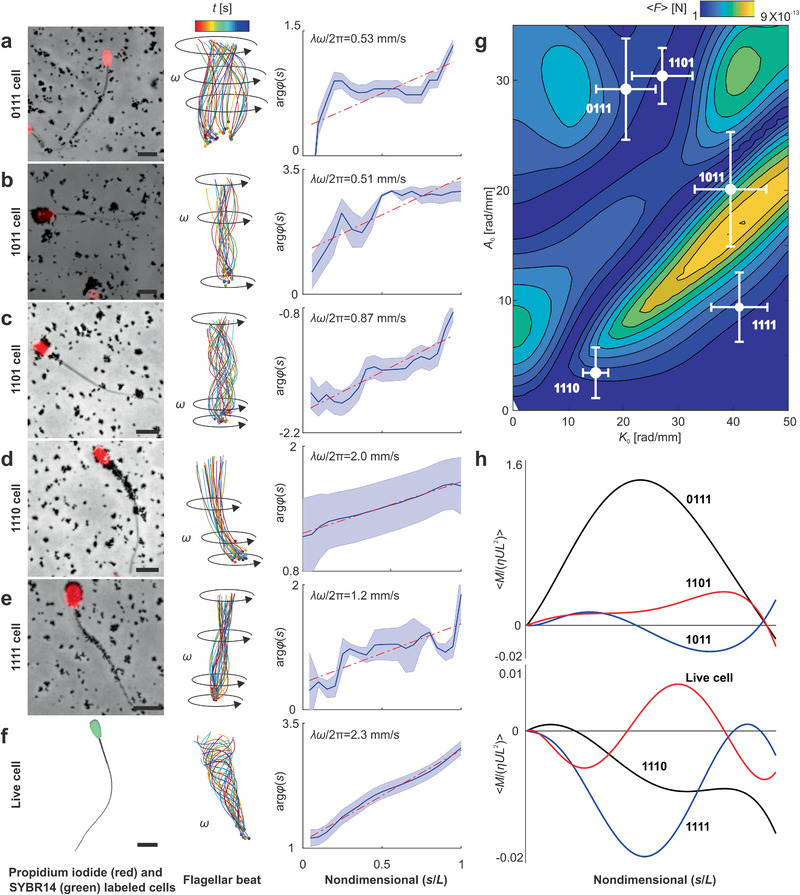
Sperm‐templated microrobots with three and four magnetic segments. a) 0111 cells. b) 1011 cells. c) 1101 cells. d) 1110 cells. e) 1111 cells. f) live sperm cell. Scale bar 10 µm. g) Time‐averaged propulsive thrust of the flagellum is calculated using RFT (see the Experimental Section) over mean flagellar curvature range of 0–50 rad mm^−1^, amplitude rise of 0–35 rad mm^−1^, and average wavelength of 394 ± 230 µm. h) The dimensionless time‐averaged bending moment 〈*M*/(*ηUL* ^2^) 〉 indicates maximum amplitude near the middle for 0111 and 1111 cells and near the distal end for 1101, 1011, 1110, and live cells. See also Video S7 (Supporting Information).

Microrobots with single and two magnetic segments along their body show different responses (Figures 4 and 5) owing to the dependence of the wave propagation direction on the position of the magnetic segment along the cell. As the number of segments with nanoparticles attached to them increases, a common behavior emerges, as shown in **Figure** **6**. Figure 6a–d indicates the rate of wave propagation along the flagellum of 0111, 1011, 1101, and 1110 cells, respectively (see also Video S7 in the Supporting Information). We observe that the transverse bending waves travel along one direction and the rates of wave propagation do not exhibit change in their sign. This uniformity in wave propagation is a consequence of the agglomerated magnetic actuation of almost all four cellular segments of the sperm cell. However, the rate of propagation along each group varies based on the amount of nanoparticles. This amount determines the induced magnetic moment once the external magnetic field is applied.

The magnetic torque exerted on each segment is quadratic in |**B**| and linear in the volume of the magnetic segment based on Equation (1). Since there is no local change in the applied field between adjacent segments, the magnetic torque depends only on the volume of the surface coatings. Figure 6g shows that maximum thrust is generated when the head, principal piece and distal ends (1011 cells) are magnetized. In this case, the time‐averaged bending moment increases toward the distal end. Associated with the increases in bending moment is an increase in the amplitude and the maximum amplitude occurs at the distal end, as shown in Figure 6h. In contrast, 0111 cells exhibit maximum bending moment near the middle of the flagellum (see Figure 6h), and this wave pattern results in near‐optimal propulsive thrust, as shown in Figure 6g. Finally, 1101 and 1011 cells exhibit lower bending moment compared with 0111 cells.

### 4.4 Sperm‐Templated Microrobots with Four Magnetic Cellular Segments

In the case of four magnetic cellular segment along 1111 cells, we also observe wave propagation along one direction, as shown in Figure 6e. Video S8 in the Supporting Information illustrates the motion of the 1111 cell versus the free motile sperm cell. We again attribute the uniformity of the wave propagation to the actuation of the four cellular segments. Despite the actuation of the four segments, we see that the rate of wave propagation is not uniform along the length compared to ATP‐driven cells, which exhibit uniform wavelength and wave speed along the flagellum, as shown in Figure 6f. The bending moment along the flagellum of 1111 cells also does not capture the characteristic of the ATP‐driven cell, as shown in Figure 6h. The maximum bending moment of the passive flagellum occurs near the middle, whereas ATP‐driven cells exhibit a gradual increase of the bending along the length. The propulsive force generated along the passive flagellum (Figures 4d, 5g, and 6g) of each microrobot is balanced by the viscous drag of the head, therefore the wave pattern of flagellum determines the swimming velocity at each actuation frequency. This wave pattern is proportional to the characteristic penetration length ℓ_*w*_ = (*κ*/*ωξ*_⊥_)^1/4^ ,^[^ ^45^ ^]^ and the amplitude of the wave pattern scales with the actuation frequency. A summary of the hydrodynamic parameters can be found in Table S1 in the Supporting Information.

## 5 Frequency Response of Sperm‐Templated Microrobots

**Figure 7:**
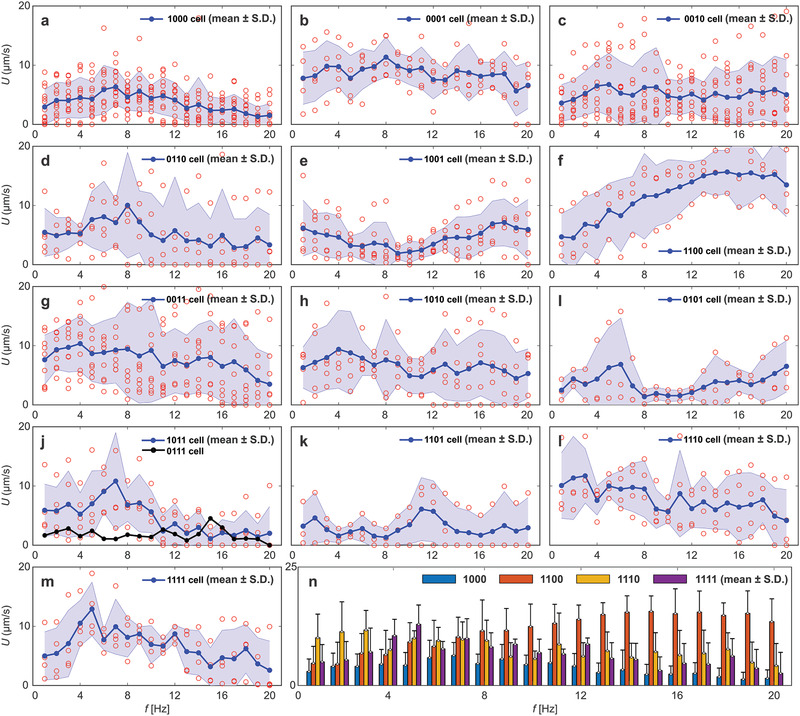
Frequency response of sperm‐templated microrobots with multiple magnetic segments along the flagellum is measured during flagellar propulsion. Average speed is measured for a frequency range of 1–20 Hz. Measured speed of a) 1000, b) 0001, c) 0010, d) 0110, e) 1001, f) 1100, g) 0011, h) 1010, i) 0101, j) 1011 and 0111 (The surface area of the head is much greater than that of any other parts of the cell making unlikely the possibility of obtaining type 0111 cells within the samples used in the frequency response experiments.), k) 1101, l) 1110, m) 1111 cells. n) Comparison between the average speeds of 1000, 1100, 1110, and 1111 cells, which achieve greatest swimming speed within the four groups.

The swimming velocity of each microrobot is measured within a frequency range of 0–20 Hz for different samples of each category, as shown in **Figure** **7**. In each trial, the microrobot is controlled to swim away from the bottom solid boundary and the average swimming speed is determined at each actuation frequency over ≈5–10 body lengths. Type 1000 cells show an increase in the swimming speed until a step‐out frequency is reached at 7 Hz. Below this frequency, the induced magnetization of the head is strong enough to enable the head to align with the rotating field and deform the flagellum. Above the actuation frequency of 7 Hz, the speed decreases with the frequency as the flagellum is not allowed to deform as much as the field rotates per unit time. 0001 and 0010 cells do not show an increase in the swimming speed with the actuation frequency. In these cases, the average swimming velocity is 8.6 ± 1.3 (*n*  =  5) and 5.2 ± 0.8 µm s^−1^ (*n*  =  10) regardless of the actuation frequency for 0001 and 0010 cells, respectively (Figure 7b,c). The response of 1000 cells (Figure 7a) and 0001 cells (Figure 7b) with a magnetic moment on either the proximal or the distal end of the cell results in a flagellar motion pattern most similar to that of motile spermatozoa.^[^ ^41^ ^]^ In these cases, the head or the distal end act as actively actuated components. The rest of the body is not covered with any particles, thus the flagellum is very flexible. However, the resulting maximum velocity is still much lower than that of live sperm (≈10 µm s^−1^ vs. 100 µm s^−1^).

Similar to type 1000 cells, the swimming velocity of microrobots with two magnetized segments at the midpiece and principal piece (0110 cells) increases until a step‐out frequency of 8 Hz to 10 ± 8.9 µm s^−1^, as shown in Figure 7d. When the magnetized segments are located at the proximal and distal ends of the cell (1001 cell) the velocity is noticeably low (2.7 ± 0.7 µm s^−1^) within the range 7 ≤ *f* ≤ 11 Hz (*n*  =  5), as shown in Figure 7e. The actuation of this type results in wave propagation along opposite directions (Figure 5b) that is not likely to yield net propulsive thrust. There is a noticeable difference in the response of 1100 and 0011 cells and the sperm‐templated microrobots with two magnetized segments. Figure 7f shows that the swimming speed of type 1100 cell increases with the actuation frequency to a maximum speed of 15.6 ± 3.6 µm s^−1^ (*n*  =  3) until a step‐out frequency of 15 Hz, while swimming velocity of type 0011 cell decreases with the frequency (Figure 7g). Here we have a strong magnetic moment at one boundary without destructively interfering propagating waves from other magnetic segments. However, when the magnetic moment is located in the middle of the cell (Figure 7c for 0010 cells and Figure 7d for 0110 cells), either on the midpiece or principal piece, the forward speed is reduced. The magnetized cellular segment near the middle is actuated and the two flexible ends (head or distal end) lag the motion of the magnetized segments, leading to propulsive thrust in opposite direction and reduced forward velocity. This is even more evident when we look at cases with two separate magnetic components (Figure 7h–k), e.g., 1010, 1101, 1011, 0101, and 1001 cells. Here, two magnetic moments are coupled with a flexible filament (the sperm body), but their actuation is counterproductive in a way that they cancel out each other's thrust. This cancellation results in average velocities below 10 µm s^−1^.

Note that the curvature of swimming path of the microrobot is proportional to the mean flagellar curvature, *K*_0_.^[^ ^42^ ^]^ We have shown that the location of the magnetized segment along the cell has a direct effect on the shape of the wave (Figures 4, 5, 6). We have also shown that the magnetization profile influences the symmetry of the flow field around the long axis of the cell (Figure 3). Therefore, the location of the magnetized segment along the cell has a direct influence on the linearity of the taken pathway. As a result, we observe a sideways shift during their forward motion (see for instance cases 1011 and 1101 in Video S7 in the Supporting Information).

Interestingly, the frequency response of the 0011 cells compared to the 1100 cells displays almost a symmetric response against the frequency (Figure 7f vs. Figure 7g). Propulsion enhancement is observed for 1100 cells with the frequency, while 0011 cells exhibit reduction in the swimming speed with the actuation frequency. The 1111 and 1110 cells (Figure 7l,m) display similar frequency response at relatively high actuation frequency, indicating that the magnetization of the distal end does not contribute much to their propulsion.

Figure 7n highlights the microrobots that outperform the rest of the configurations. The 1100 cells show noticeable propulsion enhancement, higher step‐out frequency, and improved frequency response compared to any other groups. This enhancement can be explained by the magnetization of the sperm head and midpiece (segments with greatest stiffness), leaving the flexible parts of principal piece and distal end intact for maximum flexibility. Note also that each category has a different optimal actuation frequency. For instance, 1111 cells swim at maximum speed of 12.9 ± 4.5 at a frequency of 5 Hz, whereas cells with two separate magnetic elements show maximum forward speed at frequency of 7 Hz (1011, 0110, 0101 cells). Further, 1101 cells and 1100 cells have even higher optimal frequencies at 12 and 18 Hz, respectively. Therefore, it is possible to use these distinct step‐out frequencies to separate some of the groups via dynamic excitation near the step‐out frequency of the desired group. An overview of all hydrodynamic parameters of each category can be found in the Table S1 in the Supporting Information.

It is important to note that heterogeneous behavior of the microrobots (Figure 7) enables a variety of applications that require a specific dynamic excitation. First, the diverse step‐out frequencies of each microrobot makes it possible to achieve independent motion control of multiple microrobots using a common driving magnetic field. The heterogenous response of the microrobots enables motion differentiation under a common driving field.^[^ ^46^, ^47^ ^]^ Second, microrobots with magnetized cellular segment near the middle of the flagellum have the ability to achieve bidirectional swimming inside confined environments like small vessels and channels, where it is not possible to undergo rotations or u‐turn trajectory to change the swimming direction. Figure 4c shows that transverse waves propagate along two opposite directions, enabling bidirectional swimming of 0010 cells based on the frequency of the actuating field.^[^ ^48^ ^]^ Third, the magnetized segments at the proximal and distal ends enable fluid pumping of the surrounding fluid without forward swimming. Consider, for example, the frequency response of 1001 (Figure 7e) and 0101 cells (Figure 7I) at actuation frequency of ≈10 Hz. At this frequency, these microrobots will pump the surrounding fluid (Figure 3e–I), while below and above this frequency they can move from the site of the fluid pumping. Finally, microrobots with four magnetized segments are expected to have the greatest rigidity and can undergo rotational motion, about the axis **e**_1_ × **e**_2_, and achieve fluidic trapping and noncontact micromanipulation of nonmagnetic objects.^[^ ^49^ ^]^

## 6 Discussion

Sperm‐templated microrobots created by electrostatic‐based self‐assembly of bovine sperm cells and magnetic nanoparticles open up several possibilities in soft microrobot design. The nonuniform surface charge provides an alternative method to selectively magnetize the passive elastic flagellum, and hence generates distributed bending moment. This is an important property that would enable us to design soft microrobots with distributed magnetization profiles corresponding to any desired specific motion of the flagellum. Consider, for example, the difference between a passive elastic filament driven from its proximal end (Figure 4a) and an ATP‐driven cell (Figure 6f). The normalized bending moment decreases toward the distal end in the case of actuation from the proximal end (1000 cells). In contrast, ATP‐driven cells show an increasing bending moment toward the distal end, as shown in Figure 6f. Therefore, the amplitude of 1000 cells decreases along the length of the flagellum, leading to a maximum swimming speed that is twenty times lower than that of ATP‐driven cells. Now consider the situation where the normalized bending waves increase toward the distal end, as in the case of 1100, 1110, and 1111 cells. The wave of displacement is in phase with the progressive bending moment, leading to a propagating wave with increasing amplitude toward the distal end that can be comparable to that of ATP‐driven cells. Consequently, the frequency response and the swimming speed of these configurations are improved compared to the passive flagellum driven from one end.

Our sperm‐templated microrobots are notably slower than live sperm cells. This raises the question of how we can achieve a velocity of the flexible magnetic sperm‐templated microrobots comparable to that of motile sperm. First, ATP‐driven cells have an entirely different propulsion source. The actuation is generated internally by the molecular motors along the whole length of the flagellum. We apply external magnetic torque to create a bending motion. A first requirement to approach a commensurate velocity of ATP‐driven cells with our sperm‐templated robots is to match their wave patterns. The stiffness‐frequency‐viscosity combination, defined by the characteristic penetration length $\ell_{w}$, is an important factor that governs the propulsive force. This combination also influences the resulting velocity of the microrobots and needs to be improved in further studies. The current study provides a starting point to understand and design flexible magnetic microrobots that match the performance of biological swimmers.

We have already demonstrated the influence of the location of the magnetized segment along the filament and the actuation frequency on the swimming velocity and it is evident that the magnetization profile has a direct effect on the shape of the wave, and consequently, the fluid response, and the time‐averaged propulsive thrust. The main drawback of the electrostatic self‐assembly is that the choice of the amount of nanoparticles per each cellular segment is limited by the surface charge of the cell and other factors, but to a lesser extent. The amount of nanoparticles has a direct effect on the induced magnetization based on Equation (1), and can improve the step‐out frequency of the microrobots. For example, the step‐out frequency of 1000 cells (Figure 7a) is 7 Hz, whereas 1100 cells can exhibit flagellar propulsion and trail behind the field up to 15 Hz (Figure 7f). In contrast, the step‐out frequency of 1111 cells, which are covered with more nanoparticles, is 5 Hz indicating that the amount and distribution of the magnetized segment also play a crucial role in the propulsion enhancement.

We have previously demonstrated two basic functionalities of the sperm‐templated microrobots by loading the inside of the sperm cells with anticancer drug and localization of using ultrasound imaging.^[^ ^28^ ^]^ These functionalities open up additional possibilities in translating these soft microrobots into in vivo biomedical applications. Specifically, if the inside of the cells is loaded with biochemical agents, the external magnetic field will move the microrobots in bodily fluids to achieve targeted therapy inside the body. Loading the inside of the cell with small therapeutic agents such as RNA (antisense oligonucleotides (ASOs), aptamers, siRNAs or miRNAs)^[^ ^50^ ^]^ to treat genetic and autoimmune diseases is also an area that remains to be explored. In addition to dealing with these targeted delivery applications, being primarily an essential element of fertilization, the sperm‐templated microrobots hold the greatest promise for applications in the reproductive tract as the sperm design is optimized to swim in such environment of high viscosity, fluid flow, and microarchitecture. Finally, when flexible organic bodies are combined with artificial components, new microrobot designs with advanced functionality can be expected which hold promise for applications in minimally invasive surgery, targeted drug delivery and single cell manipulation. Thus far, biohybrid microrobots have demonstrated specific potential for in vivo applications such as imaging, drug delivery and deep tissue penetration.^[^ ^6^, ^51^, ^52^, ^53^ ^]^

## 7 Conclusions

Several magnetic sperm‐templated microrobots have evolved by bio‐adhesion of magnetic nanoparticles and bovine sperm cells. They are sorted in four different groups based on the number of magnetized segments along the cell to reveal a number of optimal microrobot designs. The measured wave patterns and theoretical predictions based on the resistive‐force and regularized‐Stokeslets theories are combined to determine their fluid response, propulsive thrust, and frequency response during flagellar propulsion. The first design consists of a passive flagellum with four magnetized cellular segments leading to asymmetric transverse flow‐field, unidirectional transverse wave propagation, near‐optimal wave variables (*K*_0_,*A*_0_, and *λ*) and propulsive thrust, and relatively low step‐out frequency. A second design is that of two magnetic cellular segments on the proximal end of the cell, leading to frequency response enhancement, relatively higher step‐out frequency and near‐optimal propulsive thrust using unidirectional transverse waves. A third design consists of a single magnetized cellular segment at either end of the cell, resulting in asymmetric transverse flow fields and near‐optimal propulsive thrust. Our analysis also reveals sperm‐templated microrobot designs capable of swimming with symmetric transverse flow fields when the principal piece is magnetized, and when all segments are magnetized except for the distal end. Finally, our work shows a variety of highly compliant, biodegradable, and biocompatible microrobots. Although fabricated from a sperm template and actuated at frequencies comparable to that of live cells, their maximum swimming speed is six times lower than ATP‐driven cells.

## 8 Experimental Section

##### Fabrication and Microscopic Analysis

Sperm‐templated microrobots were fabricated by electrostatic‐based self‐assembly method.^[^ ^28^ ^]^ Bovine sperm from Holstein bulls were obtained from Masterrind GmbH Meißen and stored in liquid nitrogen. The semen straws where thawed in 37 °C water bath for 2 min, before diluting the semen in 1 mL SP‐TALP (Caisson labs). The sperm sample was centrifuged at 300 g for 5 min, the supernatant removed and resuspended in distilled water. This washing step was repeated twice before adding the elongated maghemite rice grain‐shaped nanoparticles with average diameter of 150 nm. Samples were stored at 5 °C until further use. Motile sperm were prepared in a similar way, but not resuspended in distilled water; instead, they were kept in SP‐TALP at 37 °C for the whole duration of the experiments. The viscosity of SP‐TALP at 37 °C was very similar to water, ≈1 mPa s.^[^ ^54^ ^]^ At swimming velocity of 100 µm s^−1^, Reynolds number was on the order of ≈10^−2^.

Brightfield images were obtained with a Zeiss Axiocam camera, 40x objective. For the determination of the likelihood of occurance of each category and the amount of magnetic particles of sperm‐templated microrobots, a total number of 116 images was analyzed. The observed sperm‐templated microrobots were categorized according to whether nanoparticles were attached to the head, midpiece, principal piece and/or distal end. The amount of nanoparticles was determined by measuring the particle‐covered area by the area tool in ImageJ. Then, assuming a monolayer of particles and knowing the height of each particle was hetero nm and the density of maghemite was 5.24 g cm^−3^, the total volume of nanoparticles of each cell was calculated. The fluorescent images were obtained with a Rhodamine filter of sperm‐templated microrobots incubated with red fluorescent dye propidium iodide (Live/Dead sperm viability kit, Thermo Fisher, L7011) to label the sperm heads. The cells were stained by adding 1 µL propidium iodide to 100 µL cell suspension. Cryo‐scanning electron images were taken as previously described.^[^ ^28^ ^]^

##### Numerical Simulations

The head of the cell was attached to a midpiece and connected to a flexible flagellum of length *L*, diameter 2*r*, and bending stiffness *κ*. The projected shape of the flagellum was described by the position vector of the centerline **r**(*s*, *t*) at any time *t* along the arc length *s* (0 ≤ *s* ≤ *L*). **r**(*s*, *t*) was expressed with respect to the material frame of reference of the sperm head (**e**_1_(*t*),**e**_2_(*t*)), where **e**_1_and **e**_2_ were orthonormal vectors such that **e**_1_ was oriented along the long axis of the sperm head. The centerline **r**(*s*, *t*) was characterized by the tangent angle *φ*(*s*, *t*) which was enclosed between the axis **e**_1_and the local tangent (**t**(*s*, *t*)) of the centerline of the flagellum. Nanoparticle agglomerates can adhere to the four cellular segments, creating fifteen sperm‐templated microrobots. The microrobots were magnetized by a periodic magnetic field constrained to rotate about **e**_*x*_ with angle $\theta \in [0^{\circ },90^{\circ }]$ between magnetic field and local tangent vector **t**(*s*, *t*). The field magnetizes the agglomerate to a magnetization **m**_*i*_, lying between **B** and the local tangent **t**(*s*, *t*) and the angle between **m**_*i*_ and **t** was $\varphi \in [0^{\circ },90^{\circ }]$. A rotating field at frequency of oscillation *ω* enables **m**_*i*_ to trail behind, leading to local bent at the *i*th segment along the flagellum and wave propagation. The force exerted on the fluid by the flagellum at a point *s* was proportional to the velocity of the neutral line **r**(*s*, *t*)^[^ ^55^ ^]^

(8) $$f\left(s,t\right)\;=\xi_{⊥}\;U_{⊥}\left(s,t\right)+\xi_{∥}U_{∥}\left(s,t\right),$$

where ***U***_∥_ and ***U***_⊥_ were obtained by separating $\dot{r}\left(s,t\right)$ into components lying along the directions tangential and normal to the flagellum centerline at a point *s*, respectively. The drag coefficients used for computing the force are given by^[^ ^56^ ^]^

(9) $$\xi_{∥}=\frac{2\pi \eta }{\ln\left(\frac{L}{r}\right)-0.807}\;,\;\;\;\xi_{⊥}=\frac{4\pi \eta }{\ln\left(\frac{L}{r}\right)+0.193}\;$$

Note that the relation between the magnetization of cellular segment and the magnetic field is given by, $m_{i}=\frac{1}{\mu_{0}}\;X_{a}^{i}B$, where $X_{a}^{i}$ is the susceptibility of the *i*th cellular segment and is approximated as follows^[^ ^39^ ^]^

(10) $$X_{a}^{i}=\;\mathrm{diag}\left(\frac{1}{n_{a}^{i}},\frac{1}{n_{r}^{i}},\frac{1}{n_{r}^{i}}\right)$$

where $n_{a}^{i}$ and $n_{r}^{i}$ are the demagnetization factors along the axial and radial directions of the *i*th cellular segment, respectively.

##### Fourier Analysis of the Wave Pattern

The flagellum is characterized by the tangent angle that is governed by Equations (4) and (5), and can be described by the zeroth (*φ*_0_) and first (*φ*_1_) Fourier modes as follows

(11) $$\varphi \left(s,t\right)=\varphi_{0}\;\left(s\right)+\varphi_{1}\left(s\right)e^{i\omega t}+\varphi_{1}^{*}\left(s\right)e^{-i\omega t},$$

where $\varphi_{1}^{*}\left(s\right)$ is the complex conjugate of the first Fourier mode. The zeroth mode characterizes the averaged mean shape of the magnetically actuated flagellum ( ${\tilde{\varphi }}_{0}=K_{0}\;s$), while its bending amplitude (|*φ*_1_| = *A*_0_ *s*) and wave propagation speed (arg(*φ*_1_)) are characterized by the magnitude of the complex conjugate of the first Fourier mode. The microrobots were to achieve swim and the flagellar beat patterns were measured using a microscopic unit (MF Series 176 Measuring Microscopes, Mitutoyo, Kawasaki, Japan). Videos are acquired using a camera (avA1000‐120kc, Basler Area Scan Camera, Basler AG, Ahrensburg, Germany) and a 3 × Mitutoyo phase objective. The time‐dependent deformation, **r**(*s*, *t*), is measured by discretizing the flagellum over three consecutive beat cycles at actuation frequency (*ω*  =  2*πf*) of 1 Hz. From the Fourier decomposition of **r**(*s*, *t*), the mean flagellar curvature *K*_0_, amplitude rise *A*_0_, and wavelength *λ* were determined. These parameters characterize the shape of the flagellar beat and RFT obtains the total force of the flagellum.

##### Thrust Force Calculation

The wavelength of the flagellar bending waves varies between 2.7*L* and 5.6*L* for microrobots with one and four magnetized cellular segments, respectively. Therefore, the interactions between flows induced by different parts of the flagellum can be neglected and RFT was used to predict the thrust force. The local unit tangent (**t** (*s*, *t*) = d*φ*/d*s* ) and normal vectors (**n**(*s*, *t*)) at each discretized segment were calculated along the flagellum. The velocity along the local tangent was calculated as $U_{∥}\left(s,t\right)=\left(\dot{r}\left(s,t\right)\cdot t\left(s,t\right)\right)\;t\left(s,t\right)$ and the normal velocity was calculated using $U_{⊥}\left(s,t\right)=\dot{r}\;\left(s,t\right)-U_{∥}\left(s,t\right)$. RFT predicts the thrust by integrating the local forces on each segment using Equation (6) and the drag coefficients (9). The velocity of each segment along the flagellum was determined using the reconstructed wave pattern based on the characterized mean flagellar curvature, amplitude, and wavelength.

##### Frequency Response Experiments

Sperm‐templated microrobots were suspended in water and allowed to swim under the influence of rotating magnetic fields. The magnetic field was generated using tri‐axial electromagnetic coils and the maximum magnetic field in the common center of the coils was 5 mT. The samples were observed using a microscopic unit (MF Series 176 Measuring Microscopes, Mitutoyo, Kawasaki, Japan). Videos were acquired using a camera (avA1000‐120kc, Basler Area Scan Camera, Basler AG, Ahrensburg, Germany) and a 3 × Mitutoyo phase objective. Regardless of the magnetic moment of the samples, rotating magnetic field was applied to determine the frequency response of all groups. The propulsion axis (long axis of the head) of the microrobots aligns with the rotation axis of the magnetic field. To avoid near‐surface effect on the helical propulsion, the axis of rotation of the applied magnetic field was controlled to have a nonzero pitch angle (angle with the horizontal plane). This angle enables the samples to swim upward away from the surface and from the nanoparticle aggregates. Once located away from the solid boundary of the reservoir, the helical propulsion of the microrobots was measured for frequency range of 1–20 Hz. The steering angle (angle between the magnetic field and the vertical axis) was used to control the position of microrobots within the same field of view of the microscopic system. In each trial, the samples were controlled using the pitch and steering angles of the field and the velocity was determined at each actuation frequency from a flagellar swim over 5–10 body lengths.

##### Statistical Analysis and Data Presentation

All values are presented as mean ± standard deviation (S.D.). The numbers of samples in the 1000 and 0010 cells were thirteen and eleven, respectively, and those of the samples 1100, 0011, and 0101 cells were three, nine, and three, respectively. The number of samples in the 1101 cells were four, the number of samples in 1110 and 1111 cells were four, and that of all the other groups (0001, 0110, 1001, 1010, and 1011 cells) was five. The time‐averaged velocity, 〈*U*〉, of the flow fields of all groups was normalized by the product of the length of the sperm cell and the actuation frequency, *Lf*. The distributed bending moment, *M*(*x*), of all groups was normalized by the product of the viscosity, velocity, and squared length of the cell, *ηUL* ^2^. The time‐averaged 〈*M*/*ηUL* ^2^〉 was determined for each group from a complete beat cycle based on the total number of captured frames. Each flagellar wave pattern was represented using equally‐spaced measured positions along the centerline. Polynomial regression was used to model the amplitude as a third degree function of *x* in MATLAB software (version R2017b, MathWorks, Natick, Massachusetts, United States). The tangent angle along the arc length of the determined polynomials was used in the Fourier analysis of the wave pattern to determine the average *K*_0_, *A*_0_, and *λ*.

## Conflict of Interest

The authors declare no conflict of interest.

## Supporting information

Supporting Information

Supplemental Video 1

Supplemental Video 2

Supplemental Video 3

Supplemental Video 4

Supplemental Video 5

Supplemental Video 6

Supplemental Video 7

Supplemental Video 8
