# Supplementary material for: Impact of Segmented Magnetization on the Flagellar Propulsion of Sperm‐Templated Microrobots
Source: Adv Sci (Weinh). 2021 Feb 24;8(8):2004037. doi: 10.1002/advs.202004037 (PMC8061355; doi:10.1002/advs.202004037)
Supplement: Supplementary file 1 — Supporting Information [file ADVS-8-2004037-s001.pdf]

WILEY-VCH

© 2020 Wiley-VCH GmbH

ADVANCED SCIENCE Open Access

Supporting Information

for *Adv. Sci.,* DOI: 10.1002/advs.202004037

Impact of segmented magnetization on the flagellar propulsion of sperm-templated microrobots

Veronika Magdanz, Jakopo Vivaldi, Sumit Mohanty, Anke Klingner, Marilena Vendittelli, Juliane Simmchen, Sarthak Misra, Islam S. M. Khalil**

Supporting Information

Impact of segmented magnetization on the flagellar propulsion of sperm-templated microrobots

Veronika Magdanz, Jakopo Vivaldi, Sumit Mohanty, Anke Klingner, Marilena Vendittelli, Juliane Simmchen, Sarthak Misra, Islam S. M. Khalil**

Videos:
Video S1: Flow fields over one beat cycle of sperm-templated microrobots containing one magnetic segment (1000, 0001, 0010, 0100 cell).
Video S2: Flow fields over one beat cycle of sperm-templated microrobots containing two magnetic segments (1100, 0011, 0100, 1001, 1010 and 0101 cell).
Video S3: Flow fields over one beat cycle of sperm-templated microrobots containing three magnetic segments (1101, 1011, 1110 and 0111 cell).
Video S4: Flow fields over one beat cycle of a motile sperm (left) versus a sperm-templated microrobot (1111) with all segments being magnetic (right).
Video S5: Sperm-templated microrobots with one magnetic segment.
Video S6: Sperm-templated microrobots with two magnetic segments.
Video S7: Sperm-templated microrobots with three magnetic segments.
Video S8: Free sperm cell swimming versus sperm-templated microrobot with 4 magnetic segments.

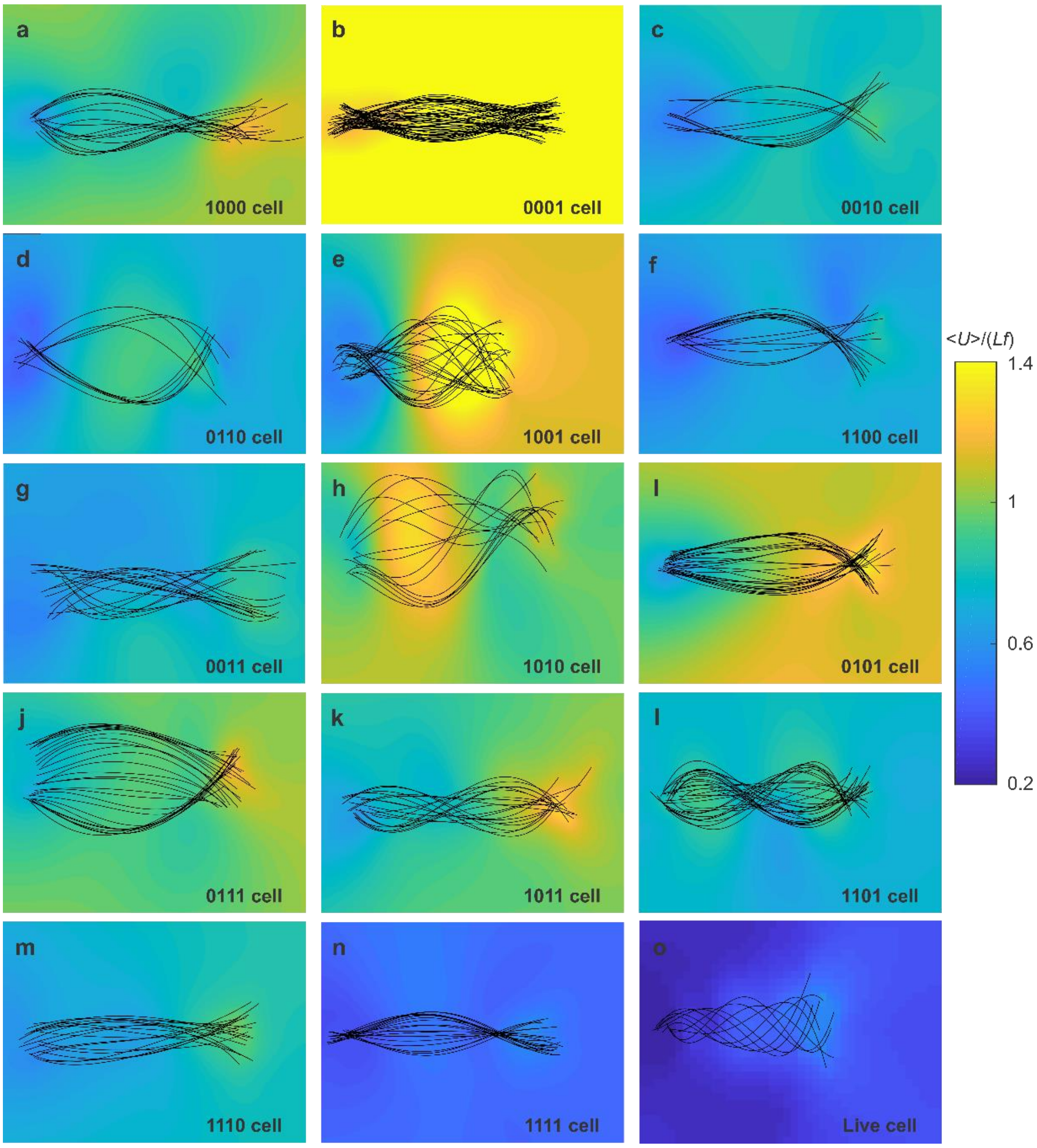

Figure S1: Average flow field (U) is calculated using the measured wave-pattern of the biohybrid microrobots for one complete beat cycle and displayed with a uniform velocity scale of 20µm/s (blue) to 120 µm/s (yellow). The wave-patterns during half a cycle (T_c) are indicated by the black lines. (**a-c**) The microrobots are actuated using single magnetic segment along the length. (**d-i**) Two magnetic segments along the length. (**j-m**) Three magnetic segments along the length. (**n**) Four magnetic segments along the length. (**o**) Flow field around a live sperm cell.

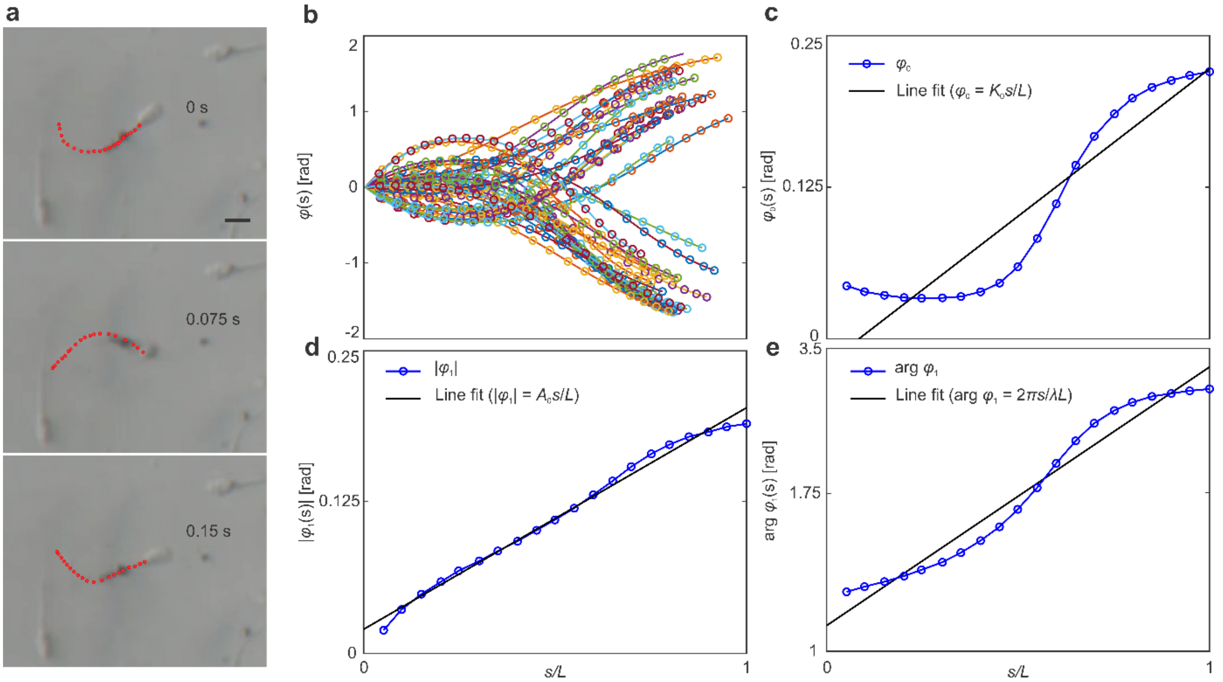

Figure S2. The instantaneous flagellar waveform is measured to determine the curvature and the bending amplitude. (**a**) A hybrid micro-bio-robot (group 0110) is allowed to swim under the influence of oscillating magnetic field at 6 Hz. The red dashed line indicates the waveform of the beating flagellum. (**b**) The tangent angle is determined based on the measured flagellar waveform. (**c**) The zeroth Fourier coefficient of the tangent angle is determined as a function of (s/L) and provide the mean flagellar curvature, K_0. (**d**) The first Fourier coefficient provide the amplitude rise, A_0. (**e**) The complex conjugate of the first Fourier coefficient provides the wavelength, λ.

Table S1: Summary of hydrodynamic parameters all categories of sperm-templated microrobots including flagellar curvature K_0, amplitude A_0, wavelength λ and maximum swimming speed $\boldsymbol{V}_{\mathbf{max}}$.

**Group**	$\mathbf{K_0}$ (rad mm^{-1})	$\mathbf{A_0}$ (rad mm^{-1})	λ (µm)	$\mathbf{V_{max}}$ (**µm** s^{-1})
1000	15.9 ± 1.2	6.1 ± 2.3	159.7 ± 15.3	6.3 ± 3.0 (f = 7 Hz)
0100	22.3 ± 4.5	4.9 ± 2.2	444 ± 258.4	5.0 ± 5.9 (f = 5 Hz)
0010	47.1 ± 5.6	2.5 ± 0.6	179.3 ± 96	6.7 ± 5.2 (f = 5 Hz)
0001	16.9 ± 2	21.1 ± 1.7	221.9 ± 4.7	11.3 ± 3.3 (f = 8 Hz)
0110	4.4 ± 0.58	17.6 ± 2.0	157 ± 31.7	10.0 ± 8.9 (f = 8 Hz)
1001	14.3 ± 5.3	27.9 ± 4.5	149.2±14.6	7.1±3.9 (f=18 Hz)
0011	5.4±1.0	9.9±3.4	434.8±160.6	10.3±4.6 (f=4 Hz)
1100	11.2±1.1	12.3±1.4	342.7±150.0	15.6±3.6 (f=15 Hz)
1010	17.0±5.3	17±6	143.4±19.2	9.4±6.5 (f=4 Hz)
0101	31.3±3.8	43.7 ± 8.3	442.2±92.0	6.8±7.8 (f=6 Hz)
0111	20.5±5.4	29.2±4.6	279.5±14.6	4.5 (f=15 Hz)

1011<br>1101<br>1110	39.5±6.5<br>27.1±5.5<br>15.0±2.29	20.1±5.2<br>30.4±2.5<br>3.4±2.3	155.4±22.4<br>414.6±33.5<br>709.5±15.9	10.8±8.1 (f=7 Hz)<br>6.1±5.3 (f=11 Hz)<br>11.7±4.5 (f=3 Hz)
1111	41.1±5.1	9.39±3.15	214.1±258.3	12.9±4.5 (f=5 Hz)
